# Association of reproductive factors and chronic kidney disease in postmenopausal women: results from the U.S. National Health and Nutrition Examination Survey 1999 - 2020

**DOI:** 10.1080/0886022X.2026.2665868

**Published:** 2026-05-13

**Authors:** Qian Wang, Rong Li, Yi-chen Liu, Juan Chang

**Affiliations:** Department of Nephrology, Chongming Hospital Affiliated to Shanghai University of Medicine and Health Sciences, Shanghai, China

**Keywords:** Chronic kidney disease, reproductive factors, restricted cubic spline, postmenopausal women

## Abstract

Chronic kidney disease (CKD) poses a growing global health burden, and reproductive history may influence CKD risk *via* hormonal pathways. We conducted a population-based cross-sectional analysis using ten cycles of the National Health and Nutrition Examination Survey (NHANES, 1999–March 2020), including 12,912 postmenopausal women with complete reproductive and kidney data. CKD was defined as estimated glomerular filtration rate <60 mL/min/1.73 m^2^ (CKD Epidemiology Collaboration 2021) or urinary albumin-to-creatinine ratio ≥30 mg/g. Survey-weighted logistic regression and restricted cubic spline models were used to examine associations between reproductive factors and CKD, adjusting for demographic and clinical confounders. After adjustment, higher parity was associated with increased CKD odds: three to four live births (OR = 1.17, 95% CI 1.01–1.34) and five or more live births (OR = 1.22, 95% CI 1.03–1.46) compared with one to two live births. Younger age at first live birth also increased risk (≤20 years: OR = 1.36, 95% CI 1.06–1.75; 21–24 years: OR = 1.35, 95% CI 1.07–1.72). In contrast, hormone replacement therapy (OR = 0.75, 95% CI 0.66–0.84) and oral contraceptive (OR = 0.79, 95% CI 0.69–0.91) use were inversely associated with CKD. Other reproductive factors, including age at menarche, menopause, reproductive span, hysterectomy, bilateral oophorectomy, and type of menopause, showed no significant associations. Restricted cubic spline analyses indicated nonlinear relationships for most continuous reproductive exposures, except age at menarche, which was approximately linear. These findings suggest reproductive history may influence kidney health in postmenopausal women and warrant further studies to clarify underlying mechanisms.

## Graphical Abstract

**Figure:**
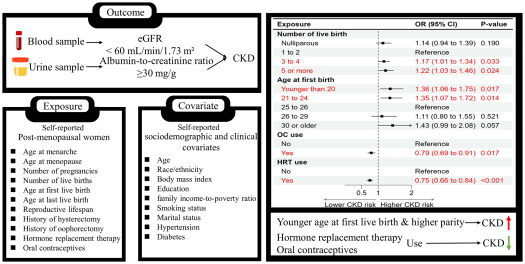

## Introduction

Chronic kidney disease (CKD) has become a major global health burden and is projected to rank as the fifth leading cause of years of life lost by 2040 [1]. Approximately 11.1% of the global population, an estimated 843.6 million individuals, is affected by CKD [2]. A growing body of evidence indicates that women tend to experience better renal outcomes than men, suggesting a potential protective role of sex hormones in renal physiology [3]. In animal models of adenine-induced nephropathy, the progression of renal injury has been shown to be slower in females than in males [4]. Similarly, premenopausal women exhibit slower CKD progression compared with men, whereas this protective effect appears to diminish after menopause [5]. In a Korean population-based study, the prevalence of CKD was 7.4% in men, 4.7% in premenopausal women, and 20.1% in postmenopausal women [6].

Compared with men, women undergo a series of natural reproductive milestones throughout their lifespan, including menarche, pregnancy, and menopause. These events substantially influence endogenous hormone levels and have long-term implications for women’s health, including cardiovascular and renal outcomes [7]. Epidemiological studies have suggested that certain aspects of reproductive history, such as age at menarche and reproductive lifespan, may be associated with CKD risk [8]. For instance, in the Korea Genome and Epidemiology Study, a longer reproductive lifespan was associated with a lower risk of CKD [9]. However, findings across studies have been inconsistent, potentially due to variations in study design, population characteristics, and the reproductive exposures assessed. In addition to these natural reproductive milestones, medical or surgical interventions, such as hysterectomy, bilateral oophorectomy, and the use of hormone replacement therapy (HRT), can also alter hormonal status and may affect kidney function. In a cohort of premenopausal women followed for a median of 14 years, those who underwent bilateral oophorectomy, particularly at or before age 45, had a higher risk of developing CKD [10]. Likewise, while some studies have reported a protective effect of HRT against albuminuria in postmenopausal women [11], others have linked HRT use to greater declines in estimated glomerular filtration rate (eGFR) [12]. In parallel, large population-based studies have demonstrated that gestational diabetes mellitus (GDM) and hypertensive disorders of pregnancy are associated with an increased risk of subsequent cardiovascular disease, CKD and even mortality [13,14]. Emerging evidence further suggests that assisted reproductive technology (ART)-related procedures themselves may influence maternal metabolic risk. For example, prolonged embryo culture duration and blastocyst transfer have been independently associated with an increased risk of gestational diabetes mellitus in IVF and frozen embryo transfer pregnancies [15,16]. However, these pregnancy-related complications have rarely been jointly considered in analyses of reproductive history and CKD.

The National Health and Nutrition Examination Survey (NHANES) is a nationally representative, cross-sectional survey conducted in the United States to assess the health and nutritional status of the civilian, noninstitutionalized population [17]. In recent years, NHANES data have been increasingly used to examine associations between lifestyle, environmental, and biological factors and a wide range of health outcomes, including CKD [18,19]. Despite this, few studies have explored the relationship between reproductive factors and CKD using NHANES data. One analysis involving 4,945 participants across eight survey cycles reported that both early natural and surgical menopause were associated with an increased risk of CKD [20]. However, population-based evaluations integrating multiple reproductive factors across the female life course remain limited.

Therefore, we conducted a cross-sectional analysis using NHANES data to investigate the associations between a broad range of reproductive milestones and interventions and the risk of CKD among U.S. women, with additional sensitivity analyses accounting for pregnancy-related metabolic complications where data were available.

## Method

### Study population

We conducted a population-based, cross-sectional analysis using ten consecutive NHANES cycles from 1999–2000 through 2017–March 2020. The most recent cycle (March 2021–March 2023) was excluded because neither the Standard Biochemistry Profile (serum creatinine, urinary albumin, and urinary creatinine) nor the Reproductive Health Questionnaire (RHQ) had been publicly released as of January 24, 2025. NHANES provides nationally representative data on health, nutrition, and related risk factors in the non-institutionalized U.S. population using a multistage, probability-based sampling design [21]. Participants completed structured interviews at home and in mobile examination centers (MECs), covering topics such as demographics and reproductive health, and provided blood and urine samples for laboratory testing. All NHANES protocols were approved by the National Center for Health Statistics (NCHS) Ethics Review Board. Specifically, the following protocols applied to the survey cycles included in this analysis: Protocol #98-12 (1999–2004), #2005-06 (2005–2010), #2011-17 (2011–2016), and #2018-01 (2017–March 2020). All participants provided written informed consent. Because this study used publicly available, de-identified NHANES data, it was considered exempt from additional institutional review. All procedures adhered to the ethical standards of the Declaration of Helsinki.

We focused on postmenopausal women who had data available for at least one reproductive factor of interest and laboratory measures required to define CKD. We restricted the analysis to postmenopausal women for two main reasons: (1) they have completed their reproductive lifespan, providing more stable reproductive history data, and (2) CKD is more prevalent among older adults [22], enhancing both the clinical relevance and statistical power of the study. Menopausal status was determined from the RHQ administered at the MECs. Participants completed the questionnaire *via* touchscreen self-interview, with trained interviewers available to assist if needed. Women were asked whether they had experienced at least one menstrual period in the past 12 months (response options: Yes, No, Refused, or Don’t Know), and if not, the reason (e.g. pregnancy, breastfeeding, medical condition, menopause, or other). Additional items asked whether participants had undergone hysterectomy or bilateral oophorectomy. Women who self-reported menopause or who had undergone bilateral oophorectomy were classified as postmenopausal and included in the analysis [23].

### Exposures

Two categories of reproductive factors were included as exposures: those directly obtained from the RHQ and those derived through calculation.

Directly obtained exposures included self-reported age at menarche (<12, 12, 13, 14, and ≥15 years), number of pregnancies (0, 1–2, 3–4, and ≥5), number of live births (nulliparous, 1–2, 3–4, and ≥5), age at first live birth (≤20, 21–24, 25–26, 27–29, and ≥30 years), age at last live birth (≤24, 25–28, 29–31, 32–34, and ≥35 years), age at menopause (<40, 40–44, 45–49, 50–54, and ≥55 years), HRT, oral contraceptive (OC) use, history of GDM (yes or no), and histories of hysterectomy and bilateral oophorectomy, along with the age at which each surgery was performed (none, <40, 40–44, 45–50, and ≥50 years). Derived exposures were defined as follows: (1) Reproductive lifespan was calculated by subtracting the age at menarche from the age at menopause (<33, 33–35, 36–38, 39–42, and ≥43 years); (2) Type of menopause was classified as natural menopause (women who did not undergo bilateral oophorectomy before menopause) or surgical menopause (women who underwent bilateral oophorectomy before the reported age of menopause); and (3) Advanced maternal age was defined as ≥35 years at the time of the first live birth (yes or no). All reproductive factors, except for binary variables, were also analyzed as continuous measures.

The categorizations and reference groups for age at menarche, age at first and last live birth, age at menopause, reproductive lifespan, type of menopause, and history and age at hysterectomy or bilateral oophorectomy were adopted from the study by Han et al. (2025) [8] to ensure consistency with prior literature. For number of pregnancies and number of live births, the category ‘1–2’ was selected as the reference group, as it represents the most common reproductive pattern among women in the general population.

### Outcome

CKD was defined according to the Kidney Disease: Improving Global Outcomes guidelines. Participants were classified as having CKD if eGFR was below 60 mL/min/1.73 m^2^ or urinary albumin-to-creatinine ratio (UACR) ≥ 30 mg/g. eGFR was estimated using the 2021 CKD Epidemiology Collaboration (CKD-EPI) equation [24]:

$$\text{eGFR}=142\times {\left(\frac{\text{Scr}}{\kappa },1\right)}^{\alpha }\times {\left(\frac{\text{Scr}}{\kappa },1\right)}^{-1.200}\times 0.9938^{\text{Age}}\times (1.012\;\text{if female})$$

Since only female participants were included, Scr represents serum creatinine (Scr) in mg/dL, the reference value (𝜅) was set at 0.7, and an adjustment factor of 1.012 was applied. The exponent 𝛼 was assigned a value of −0.241 for females. To ensure consistency across NHANES cycles, serum creatinine values were recalibrated for the 1999–2000 and 2005–2006 cycles using NHANES-specified equations. For the 1999–2000 cycle, standard Scr was derived as:

Standard serum creatinine=0.147+1.013×uncalibrated serum creatinine

For the 2005–2006 cycle, the recalibrated value was computed as:

Standard serum creatinine=−0.016+0.978×uncalibrated serum creatinine

Because NHANES is a cross-sectional survey without longitudinal follow-up, CKD was defined at a single time point based on eGFR and UACR measurements. Therefore, it was not possible to distinguish between incident and preexisting CKD cases or to exclude participants who may have had CKD prior to the survey examination.

### Covariates

Covariates included self-reported age (continuous), race/ethnicity (Hispanic, non-Hispanic White, non-Hispanic Black, and other, including multiracial), and education attainment (<high school, high school, and > high school). Race/ethnicity was included as a covariate because both reproductive factors and CKD risk have been shown to differ across racial and ethnic groups in the United States, potentially confounding the observed associations [25]. The family income-to-poverty ratio (FIPR) was used as an indicator of socioeconomic status. The FIPR is calculated by dividing a family’s total income by the poverty threshold specific to their family size, year, and state, as defined by the U.S. Census Bureau. In this study, FIPR was categorized as <1.3 (below poverty), 1.3–3.5 (middle income), and ≥3.5 (high income), consistent with prior NHANES analyses [26]. Smoking status was classified as never smoker (individuals who had not smoked 100 cigarettes in their lifetime and do not currently smoke), former smoker (those who smoked ≥100 cigarettes in their lifetime but do not currently smoke), and current smoker (those who smoked ≥100 cigarettes in their lifetime or currently smoke). Marital status was categorized as living with someone (married or living with a partner), living alone (widowed, divorced, or separated), or never married. Hypertension was defined as a history of physician-diagnosed hypertension, a measured average systolic blood pressure ≥140 mmHg, diastolic blood pressure ≥90 mmHg, or current use of anti-hypertensive medication. Diabetes was defined as a history of physician-diagnosed diabetes, hemoglobin A1c ≥6.5%, or current use of diabetes medication.

### Statistical analyses

We first performed descriptive analyses to compare sociodemographic characteristics and comorbidities between women with and without CKD. Continuous variables were summarized as survey-weighted means with 95% confidence intervals (CIs), and categorical variables as survey-weighted proportions with corresponding 95% CIs.

Separate analyses were conducted for each reproductive exposure, using the same set of covariates and CKD as the outcome. Participants with missing data for the reproductive exposure of interest or CKD status were excluded from the corresponding analysis. Continuous covariates contained no missing values. For categorical covariates, missing values were handled by creating a distinct ‘missing’ category, allowing us to retain the maximum possible sample size for each exposure–outcome model.

To estimate the independent associations between reproductive factors and CKD, we used survey-weighted logistic regression models that accounted for the complex sampling design of NHANES. Odds ratios (ORs) with 95% CIs were reported for both crude and adjusted models. The crude models included only the exposure of interest, while the adjusted models controlled for age, BMI, race/ethnicity, educational attainment, FIPR, marital status, smoking status, hypertension, and diabetes. To examine potential nonlinear associations between continuous reproductive exposures and CKD, we fitted survey-weighted restricted cubic spline (RCS) regression models with four knots placed at recommended percentiles of the exposure distributions. Global and nonlinearity Wald tests were used to evaluate the overall and nonlinear associations. All statistical analyses were performed using R software (version 4.4.3; R Foundation for Statistical Computing, Vienna, Austria).

Sensitivity analyses were performed to evaluate the potential influence of pregnancy complications on the association between parity and CKD. GDM was analyzed both as an independent reproductive exposure and, separately, included as an additional covariate in multivariable models assessing the parity–CKD association. Because information on GDM was available only in selected NHANES cycles (2007–2020), these sensitivity analyses were restricted to participants with available GDM data. Results were therefore interpreted with consideration of the reduced sample size and statistical power. Within this restricted sample, parity was modeled with and without additional adjustment for GDM to assess the robustness of the observed parity–CKD association to inclusion of pregnancy-related complications.

## Results

### Study population characteristics

**Figure 1.**
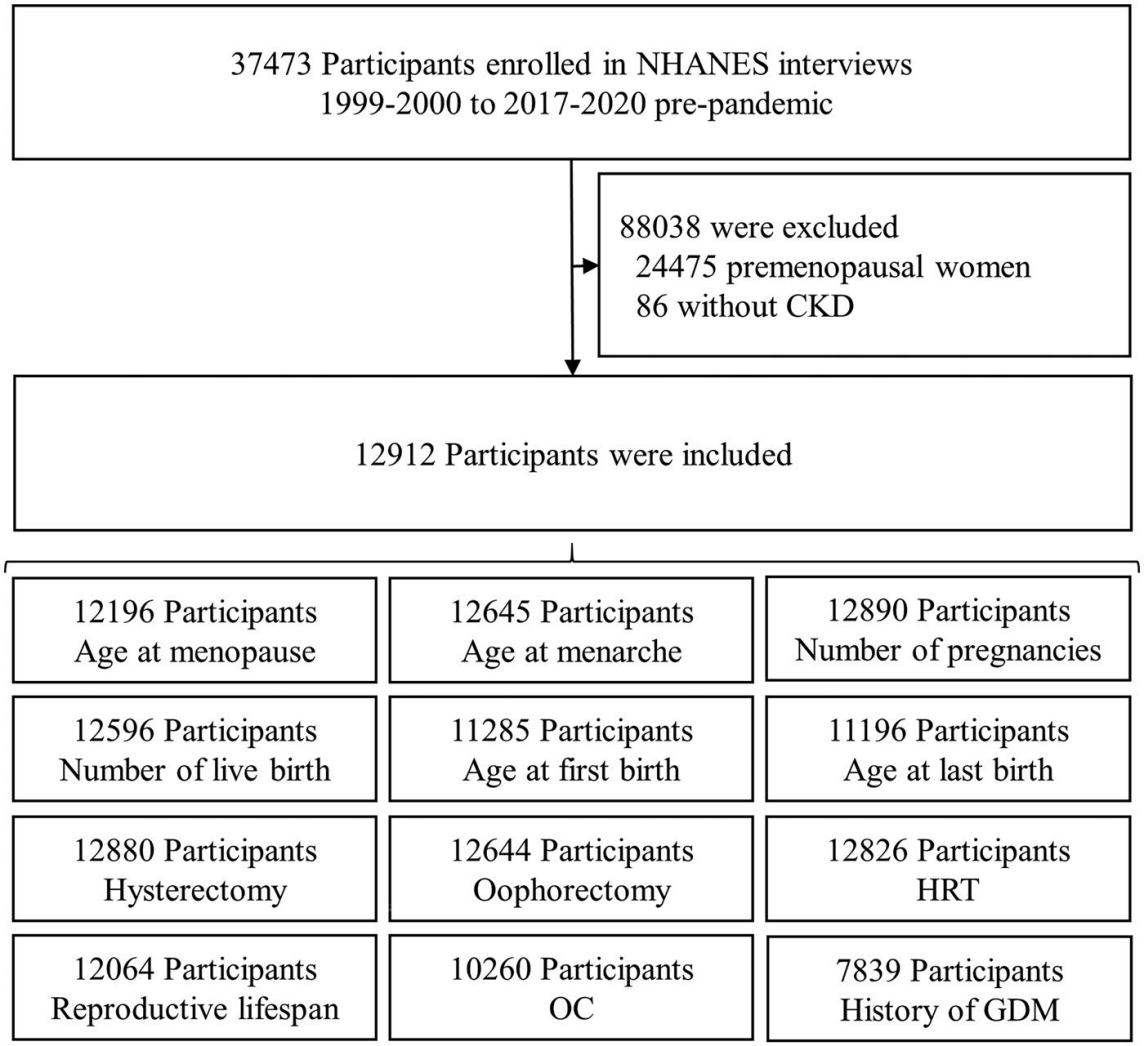
The flowchart of participants’ section progress.

A total of 37,473 participants completed the RHQ across ten NHANES cycles from 1999 to March 2020. We excluded 24,475 participants who reported having regular menstrual periods or indicated that their menopausal status was due to pregnancy, breastfeeding, or usually irregular periods. An additional 86 participants were excluded due to missing data on CKD. After these exclusions, 12,912 postmenopausal women were included in the final analysis, of whom 3,508 were classified as having CKD and 9,404 did not have CKD (Figure 1). Participants with CKD were generally older than those without CKD (69.7 vs. 60.5 years), more likely to be non-Hispanic Black, and more often living alone (Table 1). They also had lower educational attainment and lower FIPR. Specifically, 26.4% of participants with CKD had a FIPR ≤1.3 (below poverty) compared with 16.4% of those without CKD, while 25.6% of participants with CKD had a FIPR ≥3.5 (high income) versus 41.8% among those without CKD (Table 1). Participants with CKD were more likely to have hypertension (69.8% vs. 45.0%), diabetes (26.9% vs. 11.1%), and a BMI ≥30 (42.8% vs. 38.8%). Regarding smoking status, participants with CKD were more frequently former smokers, whereas those without CKD were more often current smokers. The prevalence of a self-reported history of GDM was low and comparable between women with and without CKD (Table 2).

**Table 1.** Participant characteristics by diagnosis of chronic kidney disease, national health and nutrition Examination survey (NHANES), 1999–March 2020.

	Chronic kidney disease		
Participant characteristic^⸿^	Yes (*n* = 3508)^a^	No (*n* = 9404)^a^	*P-value*
Age at sample collection^&^	69.7 (69.2, 70.2)	60.5 (60.1, 60.8)	**<0.001**
Race/Ethnicity^⁋^			
Hispanic	7.8 (6.2, 9.4)	9.2 (7.9, 10.5)	**<0.001**
Non-Hispanic White	73.0 (70.5, 75.6)	76.0 (74.0, 77.9)	
Non-Hispanic Black	14.0 (12.2, 15.8)	9.2 (8.1, 10.3)	
Other	5.2 (4.2, 6.1)	5.6 (4.8, 6.3)	
Marital status^⁋^			
Living with partner	42.7 (40.2, 45.2)	60.8 (59.2, 62.3)	**<0.001**
Living alone	51.8 (49.4, 54.3)	32.8 (31.4, 34.1)	
Never married	4.5 (3.7, 5.3)	5.5 (4.8, 6.2)	
Education^⁋^			
<High school	27.5 (25.4, 30.9)	16.2 (15.2, 17.3)	**<0.001**
High school	29.1 (27.2, 33.7)	27.0 (25.8, 28.3)	
>High school	43.2 (40.5, 45.9)	56.7 (55.1, 58.2)	
Family income-to-poverty ratio^⁋^			
≤1.3	26.4 (24.2, 28.6)	16.4 (15.0, 17.7)	**<0.001**
>1.3–3.5	40.1 (37.9, 42.3)	33.7 (32.3, 35.1)	
>3.5	25.6 (23.2, 27.9)	41.8 (39.9, 43.7)	
Smoking status^⁋^			
Never smoker	57.0 (54.8, 59.2)	55.7 (54.2, 57.3)	**<0.001**
Former smoker	30.1 (28.1, 32.1)	26.9 (25.4, 28.3)	
Current smoker	12.9 (11.3, 14.5)	17.3 (16.1, 18.6)	
Body mass index^⁋^			
<25	26.6 (24.7, 28.5)	28.7 (27.5, 30.0)	**<0.001**
25–<30	27.8 (26.1, 29.5)	31.5 (30.2, 32.9)	
≥30	42.8 (40.7, 44.9)	38.8 (37.4, 40.2)	
Hypertension^⁋^			
Yes	69.8 (67.7, 71.8)	45.0 (43.6, 46.4)	**<0.001**
No	30.0 (28.0, 32.1)	54.9 (53.5, 56.3)	
Diabetes^⁋^			
Yes	26.9 (24.9, 28.9)	11.1 (10.2, 12.0)	**<0.001**
No	70.0 (68.0, 72.0)	86.3 (85.3, 87.2)	

^a^ Unweighted sample size.

^⸿^ Weighted percentage using NHANES sampling design.

^&^ Continuous variables are presented as weighted means and 95% confidence intervals.

^⁋^ Categorical variables are expressed as weighted percentages with 95% confidence intervals.

Age at sample collection was a continuous variable with no missing values. Missing data for categorical covariates were as follows: race/ethnicity (0%), family income-to-poverty ratio (8.7%), education (0.1%), marital status (0.9%), body mass index (1.8%), smoking status (*n* = 13; 0.1%), diabetes (2.6%), and hypertension (0.2%). Missing values for categorical variables were retained as a separate “missing” category in regression analyses to preserve sample size.

**Table 2.** Crude and adjusted generalized linear regression model of the associations between categorical reproductive exposures and chronic kidney disease.

		Prevalence of reproductive exposure^⸿^		Crude model		Adjusted model^&^	
Reproductive exposures	Categories	CKD group	Non-CKD group	OR (95%CI)	*P-Value*	OR (95%CI)	*P-Value*
Age at menarche	<12	17.4 (15.6, 19.2)	20.7 (19.5, 22.0)	0.86 (0.73, 1.01)	0.069	0.88 (0.73, 1.05)	0.145
	12	26.6 (24.8, 28.4)	26.5 (25.1, 27.9)	1.03 (0.90, 1.16)	0.694	1.06 (0.91, 1.23)	0.451
	13	25.0 (23.2, 26.9)	25.6 (24.2, 27.0)	Ref.		Ref.	
	14	14.5 (12.8, 16.1)	13.3 (12.3, 14.2)	1.11 (0.93, 1.34)	0.249	1.10 (0.89, 1.35)	0.396
	≥15	16.5 (14.6, 18.4)	13.9 (12.9, 14.8)	1.22 (1.02, 1.46)	**0.033**	1.10 (0.89, 1.35)	0.382
Age at menopause	<40	23.6 (21.6, 25.5)	25.1 (23.7, 26.6)	0.91 (0.77, 1.07)	0.249	1.05 (0.89, 1.26)	0.545
	40−44	14.6 (12.8, 16.4)	15.5 (14.4, 16.6)	0.91 (0.75, 1.11)	0.359	0.88 (0.71, 1.08)	0.224
	45−49	21.8 (20.0, 23.6)	23.2 (22.0, 24.3)	0.91 (0.79, 1.05)	0.192	0.91 (0.76, 1.08)	0.262
	50−54	27.2 (24.9, 29.4)	26.3 (24.9, 27.7)	Ref.		Ref.	
	≥55	12.9 (11.1, 14.6)	9.9 (9.0, 10.8)	1.26 (1.01, 1.56)	**0.036**	0.99 (0.78, 1.24)	0.897
Number of pregnancies	0	8.2 (7.2, 9.3)	9.1 (8.3, 10.0)	1.08 (0.90, 1.30)	0.417	0.98 (0.84, 1.14)	0.777
	1-2	28.8 (26.8, 30.8)	34.5 (33.1, 36.0)	Ref.		Ref.	
	3–4	35.9 (33.9, 37.9)	37.7 (36.3, 39.0)	1.14 (1.00, 1.30)	0.043	1.14 (0.95, 1.38)	0.152
	≥5	27.0 (25.0, 29.1)	18.7 (17.7, 19.7)	1.74 (1.50, 2.01)	**<0.001**	1.09 (0.89, 1.34)	0.409
Number of live births	0	8.2 (7.2, 9.3)	9.1 (8.3, 10.0)	1.14 (0.96, 1.36)	0.141	1.14 (0.94, 1.39)	0.19
	1−2	28.8 (26.8, 30.8)	34.5 (33.1, 36.0)	Ref.		Ref.	
	3−4	35.9 (33.9, 37.9)	37.7 (36.3, 39.0)	1.49 (1.32, 1.68)	**<0.001**	1.17 (1.01, 1.34)	**0.033**
	≥5	27.0 (25.0, 29.1)	18.7 (17.7, 19.7)	2.48 (2.15, 2.87)	**<0.001**	1.22 (1.03, 1.46)	**0.024**
Age at first live birth	≤20	42.5 (39.8, 45.1)	38.2 (36.5, 39.9)	1.52 (1.21, 1.90)	**<0.001**	1.36 (1.06, 1.75)	**0.017**
	21−24	32.9 (30.8, 35.0)	29.6 (28.2, 30.9)	1.52 (1.23, 1.87)	**<0.001**	1.35 (1.07, 1.72)	**0.014**
	25−26	7.7 (6.5, 9.0)	10.5 (9.5, 11.5)	Ref.		Ref.	
	27−29	8.2 (6.7, 9.6)	11.0 (9.9, 12.1)	1.01 (0.41, 1.36)	0.933	1.11 (0.80, 1.55)	0.521
	≥30	8.8 (7.0, 10.6)	10.8 (9.5, 12.0)	1.11 (0.81, 1.53)	0.51	1.43 (0.99, 2.08)	0.057
Age at last live birth	≤24	22.0 (19.8, 24.2)	23.8 (22.3, 25.3)	1.03 (0.85, 1.24)	0.765	1.11 (0.89, 1.37)	0.35
	25−28	22.8 (20.4, 25.3)	25.4 (24.1, 26.7)	Ref.		Ref.	
	29−31	16.3 (14.6, 18.1)	18.3 (17.0, 19.5)	1.00 (0.82, 1.21)	0.623	0.91 (0.72, 1.14)	0.404
	32−34	13.7 (11.9, 15.4)	13.1 (12.0, 14.3)	1.16 (0.94, 1.42)	0.495	1.07 (0.85, 1.36)	0.553
	≥35	25.1 (22.7, 27.5)	19.3 (18.0, 20.6)	1.45 (1.20, 1.74)	**<0.001**	1.07 (0.84, 1.35)	0.581
Reproductive lifespan	<33	43.8 (41.1, 46.4)	45.3 (43.5, 47.1)	Ref.		Ref.	
	33–35	12.8 (11.2, 14.5)	13.2 (12.2, 14.2)	1.00 (0.84, 1.20)	0.963	0.97 (0.79, 1.18)	0.751
	36–38	17.9 (16.2, 19.6)	17.7 (16.6, 18.8)	1.05 (0.89, 1.24)	0.585	1.01 (0.84, 1.21)	0.914
	39–42	16.2 (14.4, 18.1)	16.4 (15.2, 17.6)	1.02 (0.87, 1.21)	0.788	0.96 (0.79, 1.17)	0.696
	≥43	9.3 (7.8, 10.7)	7.4 (6.5, 8.2)	1.30 (1.04, 1.62)	**0.021**	1.00 (0.77, 1.30)	0.991
Advanced maternal age	No	98.0 (97.3, 98.8)	96.6 (95.9, 97.2)	Ref.		Ref.	
	Yes	2.0 (1.2, 2.7)	3.4 (2.8, 4.1)	0.57 (0.36, 0.89)	0.014	0.77 (0.47, 1.28)	0.313
Types of menopauses	Natural	83.2 (81.7, 84.8)	82.1 (81.1, 83.2)	Ref.		Ref.	
	Surgical	16.8 (15.2, 18.3)	17.9 (16.8, 18.9)	0.93 (0.81, 1.06)	0.273	0.89 (0.76, 1.03)	0.124
Hysterectomy	No	53.8 (51.5, 56.1)	54.1 (52.6, 55.6)	Ref.		Ref.	
	Yes	46.2 (43.9, 48.5)	45.9 (44.4, 47.4)	1.01 (0.91, 1.12)	0.831	0.90 (0.80, 1.01)	0.073
Age at hysterectomy	None	58.7 (56.4, 61.0)	58.9 (59.3, 60.4)	Ref.		Ref.	
	<40	17.7 (15.9, 19.4)	19.9 (18.4, 21.3)	0.89 (0.76, 1.04)	0.145	1.03 (0.87, 1.23)	0.711
	40−44	7.0 (5.9, 8.2)	8.7 (7.9, 9.5)	0.81 (0.66, 1.00)	**0.048**	0.77 (0.62, 0.97)	**0.024**
	45−49	6.9 (5.8, 8.0)	6.4 (5.7, 7.1)	1.08 (0.87, 1.34)	0.468	0.91 (0.70, 1.16)	0.432
	≥50	9.7 (8.4, 11.0)	6.2 (5.6, 6.7)	1.58 (1.32, 1.89)	**<0.001**	0.94 (0.78, 1.14)	0.546
Bilateral oophorectomy	No	73.9 (72.0, 75.9)	75.8 (74.6, 77.0)	Ref.		Ref.	
	Yes	26.1 (24.1, 28.0)	24.2 (23.0, 25.4)	1.10 (0.98, 1.24)	0.108	0.96 (0.84, 1.10)	0.549
Age at bilateral oophorectomy	None	75.6 (73.7, 77.5)	77.1 (75.9, 78.3)	Ref.		Ref.	
	<40	8.0 (6.9, 9.1)	8.9 (8.0, 9.9)	0.91 (0.77, 1.09)	0.308	0.97 (0.79, 1.19)	0.762
	40−44	4.8 (3.7, 5.8)	4.8 (4.1, 5.4)	1.01 (0.77, 1.35)	0.919	0.94 (0.71, 1.26)	0.681
	45−49	3.8 (3.0, 4.7)	3.8 (3.3, 4.4)	1.02 (0.76, 1.37)	0.9	0.87 (0.63, 1.21)	0.414
	≥50	7.8 (6.6, 8.9)	5.3 (4.7, 6.0)	1.48 (1.21, 1.81)	**<0.001**	1.04 (0.84, 1.27)	0.775
HRT use	No	63.3 (61.3, 65.3)	54.7 (53.3, 56.2)	Ref.		Ref.	
	Yes	36.7 (34.7, 38.7)	45.3 (43.8, 46.7)	0.70 (0.64, 0.77)	**<0.001**	0.75 (0.66, 0.84)	**<0.001**
OC use	No	54.0 (51.3, 56.8)	30.1 (28.7, 31.6)	Ref.		Ref.	
	Yes	46.0 (43.2, 48.7)	69.9 (68.4, 71.3)	0.37 (0.33, 0.41)	**<0.001**	0.79 (0.69, 0.91)	**0.017**
History of GDM	No	5.6 (4.6, 6.8)	94.3 (93.3, 95.4)	Ref.		Ref.	
	Yes	5.3 (3.9, 6.6)	94.7 (93.4, 96.1)	0.85 (0.61, 1.18)	0.333	1.17 (0.79, 1.73)	0.427

^⸿^ Weighted percentage using NHANES sampling design.

^&^ Adjusted model includes adjustments for age, body mass index, race/ethnicity, educational attainment, family income-to-poverty ratio, marital status, smoking status, hypertension, and diabetes.

CKD: chronic kidney disease; OC: Oral contraceptives; CI: confidence intervals; GDM: gestational diabetes mellitus; HRT: Hormone replacement therapy; OR: odds ratio.

### Reproductive exposures and CKD prevalence

**Figure 2.**
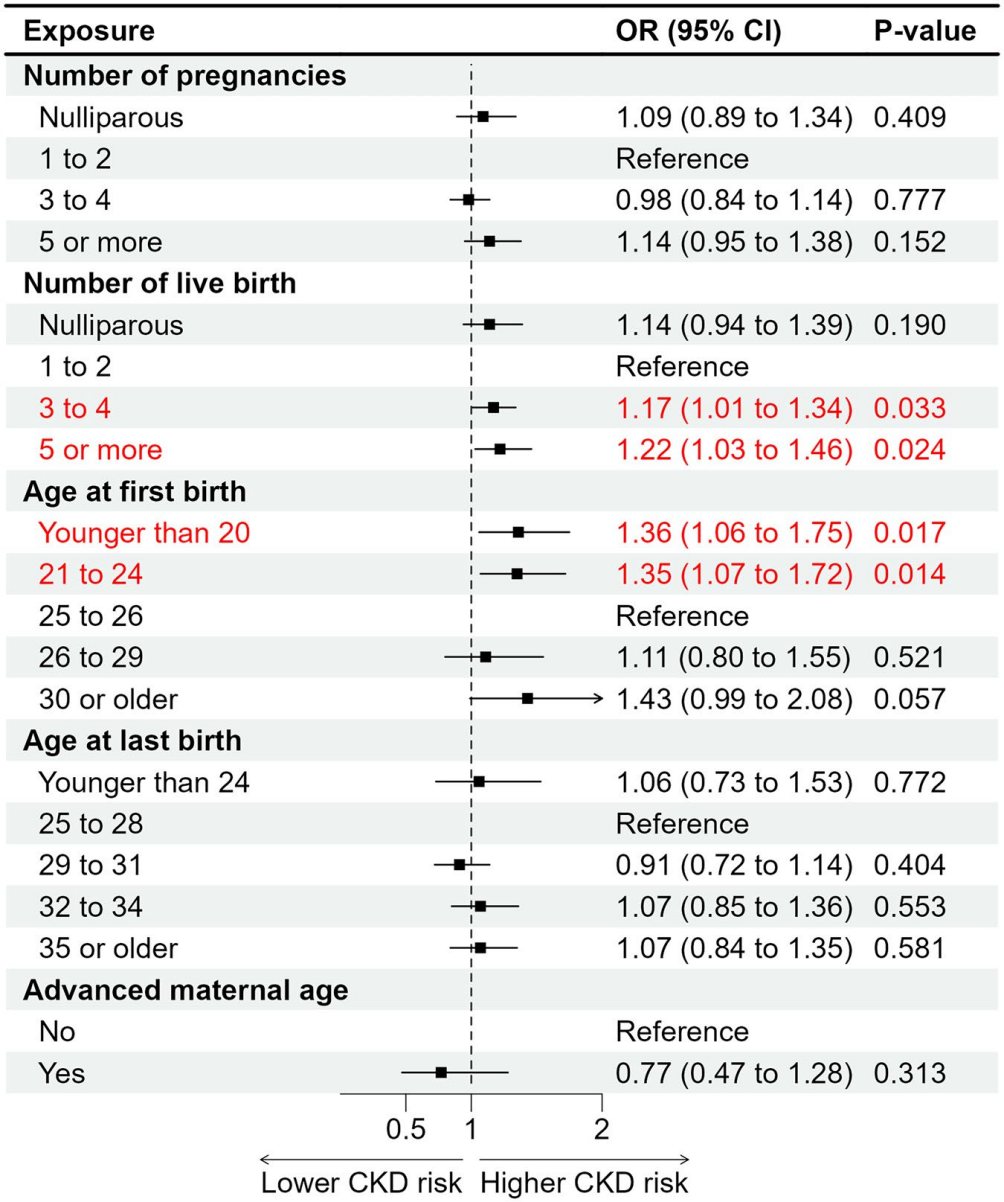
Associations between pregnancy history and childbirth characteristic with chronic kidney disease among postmenopausal women in the U.S. NHANES 1999–2020.

**Figure 3.**
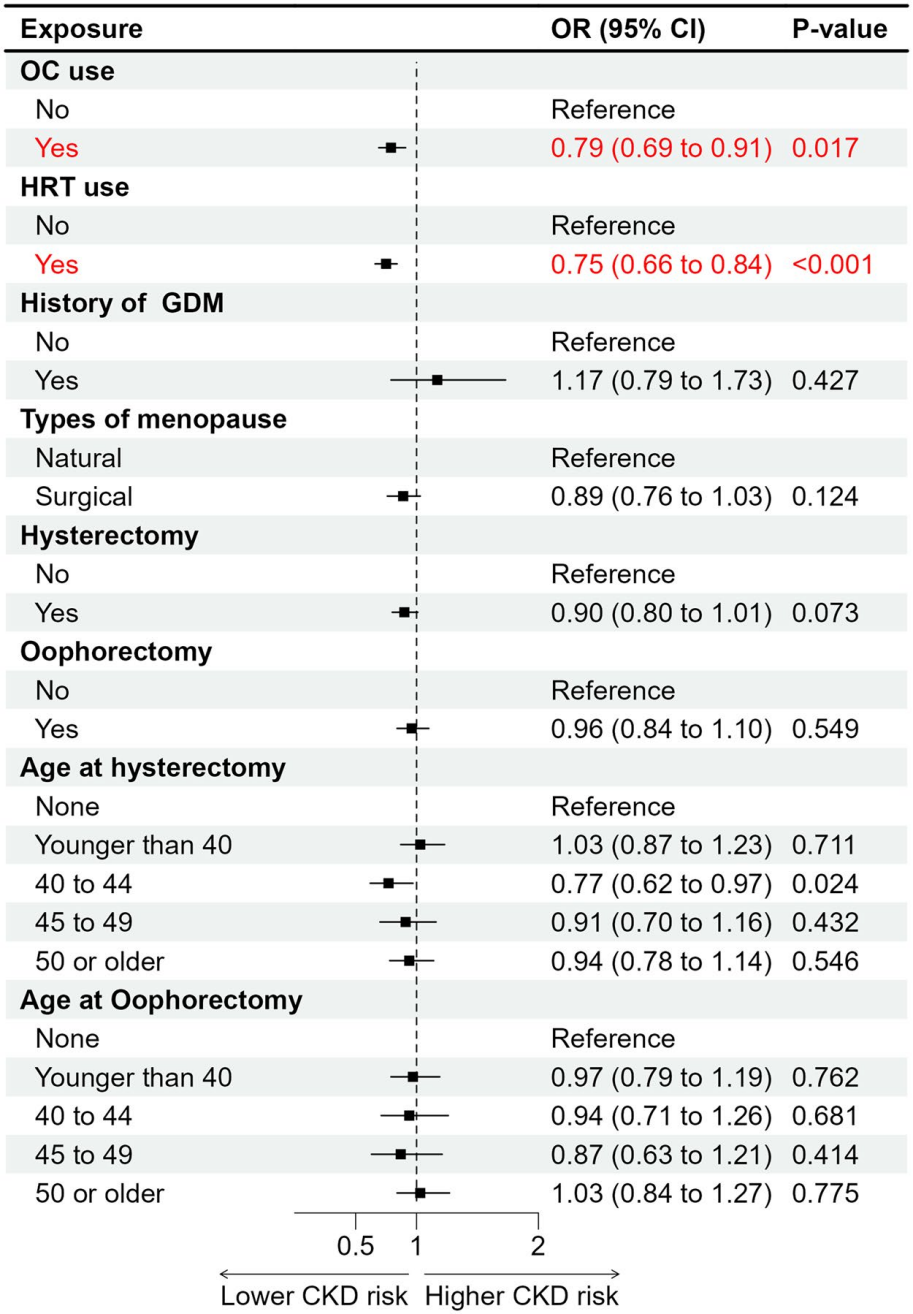
Associations between oral contraceptive (OC) use, hormone replacement therapy (HRT), history of gestational diabetes mellitus (GDM), menopause type, and gynecologic surgery history with chronic kidney disease among postmenopausal women from NHANES 1999–2020.

**Figure 4.**
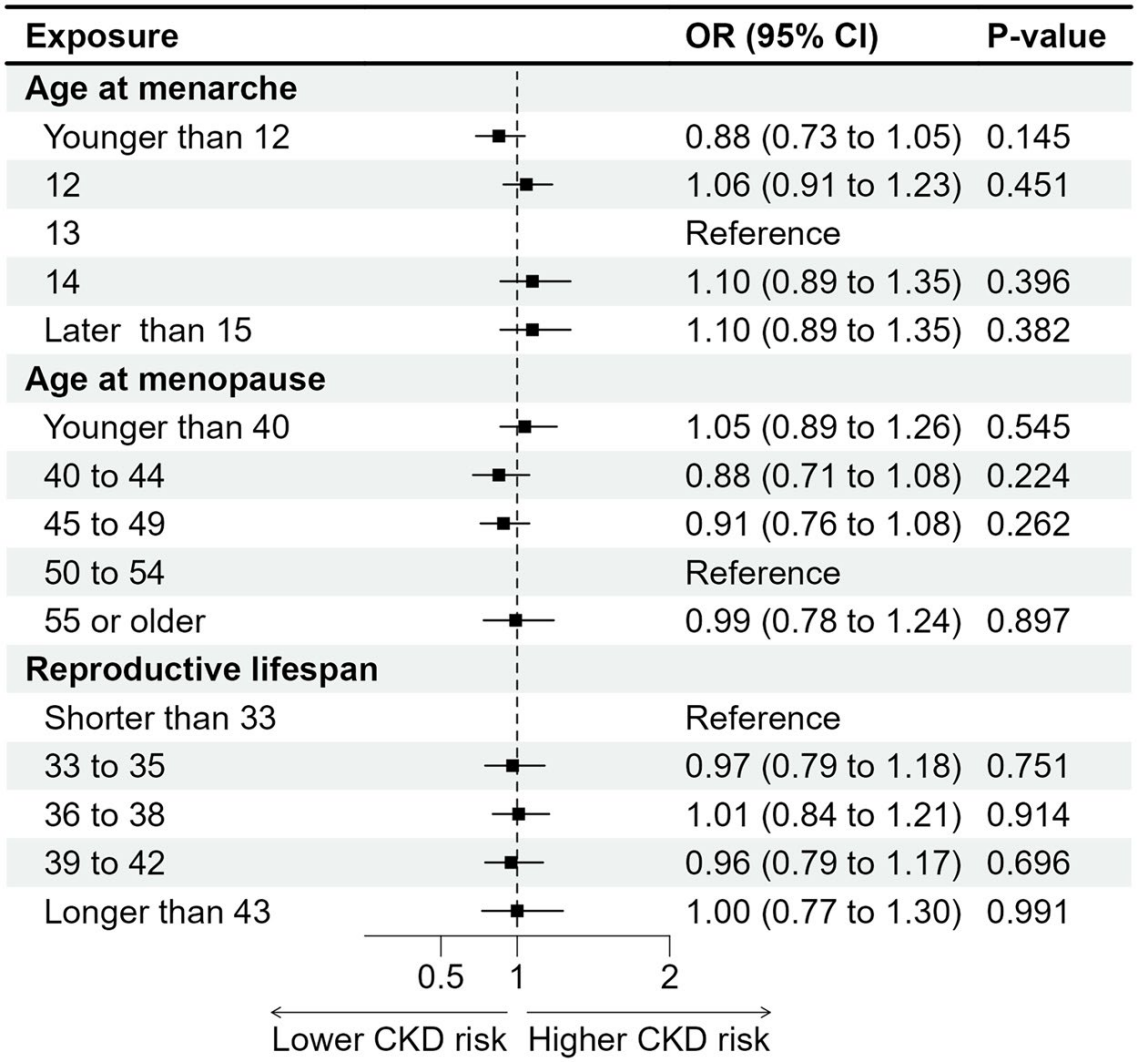
Associations between age at menarche, age at menopause, and reproductive lifespan with chronic kidney disease among postmenopausal women in the U.S. NHANES 1999–2020 cohort.

After full adjustment for potential confounders, women with higher parity showed an increased likelihood of CKD; those with three to four live births (OR = 1.17, 95% CI 1.01–1.34, *p* = 0.033) and five or more live births (OR = 1.22, 95% CI 1.03–1.46, *p* = 0.024) had significantly higher odds of CKD compared with women with one to two live births (Table 2 and Figure 2). Younger age at first live birth was also associated with elevated CKD risk. Compared with women whose first live birth occurred at 25–26 years, those with first births at ≤20 years (OR = 1.36, 95% CI 1.06–1.75, *p* = 0.017) and at 21–24 years (OR = 1.35, 95% CI 1.07–1.72, *p* = 0.014) had higher odds of CKD (Table 2 and Figure 2). In contrast, HRT use was inversely associated with CKD (OR = 0.75, 95% CI 0.66–0.84, *p* < 0.001), suggesting a potential protective effect. Similarly, OC use was associated with lower CKD prevalence (OR = 0.79, 95% CI 0.69–0.91, *p* = 0.017) (Table 2 and Figure 3). Other reproductive factors, including age at menarche, age at menopause, reproductive span, hysterectomy, bilateral oophorectomy, type of menopause, and history of GDM, were not significantly associated with CKD after adjustment (Table 2 and Figures 2–4).

**Figure 5.**
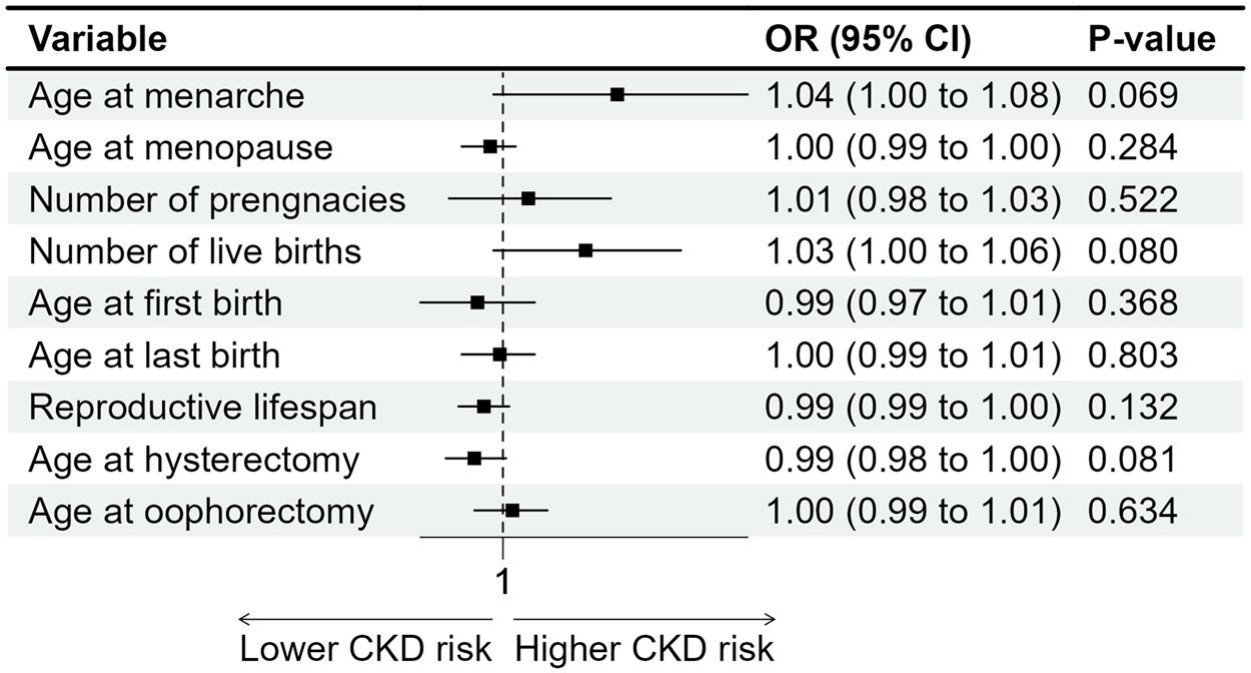
Associations between continuous reproductive exposures and the prevalence of chronic kidney disease.

For the associations between continuous reproductive exposures and CKD, older age at menarche, older age at menopause, higher numbers of pregnancies and live births, younger age at first live birth, older age at last live birth, and older ages at hysterectomy or bilateral oophorectomy were all significantly associated with higher CKD prevalence in the crude models (Table 3 and Figure 5). However, these associations were attenuated and lost statistical significance after full adjustment for potential confounders.

**Table 3.** Crude and adjusted generalized linear regression model of the associations between continuous reproductive exposures and chronic kidney disease.

	Prevalence of reproductive exposure^⁋^		Crude model		Adjusted model&	
Reproductive exposures	CKD group	Non-CKD group	OR (95%CI)	*P*-Value	OR (95%CI)	*P*-Value
Age at menarche	12.9 (12.8, 13.0)	12.7 (12.6, 12.8)	1.064 (1.032, 1.097)	**0.001**	1.036 (0.997, 1.077)	0.069
Age at menopause	45.2 (44.8, 45.7)	44.7 (44.4, 45.0)	1.007 (1.000, 1.014)	0.036	0.996 (0.987, 1.004)	0.284
Number of pregnancies	3.5 (3.4, 3.6)	3.0 (2.9, 3.1)	1.090 (1.065, 1.115)	**<0.001**	1.008 (0.983, 1.034)	0.522
Number of live births	2.9 (2.8, 3.0)	2.4 (2.3, 2.5)	1.174 (1.147, 1.202)	**<0.001**	1.026 (0.997, 1.056)	0.08
Age at first live birth	22.2 (21.9, 22.5)	22.9 (22.7, 23.1)	0.971 (0.957, 0.985)	**<0.001**	0.992 (0.974, 1.010)	0.368
Age at last live birth	29.8 (29.4, 30.1)	29.0 (28.7, 29.2)	1.022 (1.012, 1.032)	**<0.001**	0.999 (0.987, 1.010)	0.803
Reproductive lifespan	32.3 (31.9, 32.8)	32.0 (31.7, 32.3)	1.005 (0.998, 1.012)	0.185	0.994 (0.986, 1.002)	0.132
Age at hysterectomy	42.2 (41.4, 43.1)	40.3 (39.9, 40.8)	1.019 (1.010, 1.029)	**<0.001**	0.991 (0.982, 1.001)	0.081
Age at bilateral oophorectomy	44.9 (44.0, 45.8)	42.8 (42.2, 43.4)	1.021 (1.011, 1.031)	**<0.001**	1.003 (0.991, 1.014)	0.634

^&^ Adjusted model includes adjustments for age, body mass index, race/ethnicity, educational attainment, family income-to-poverty ratio, marital status, smoking status, hypertension, and diabetes.

^⁋^ Continuous variables are presented as weighted means with 95% confidence intervals.

**Figure 6.**
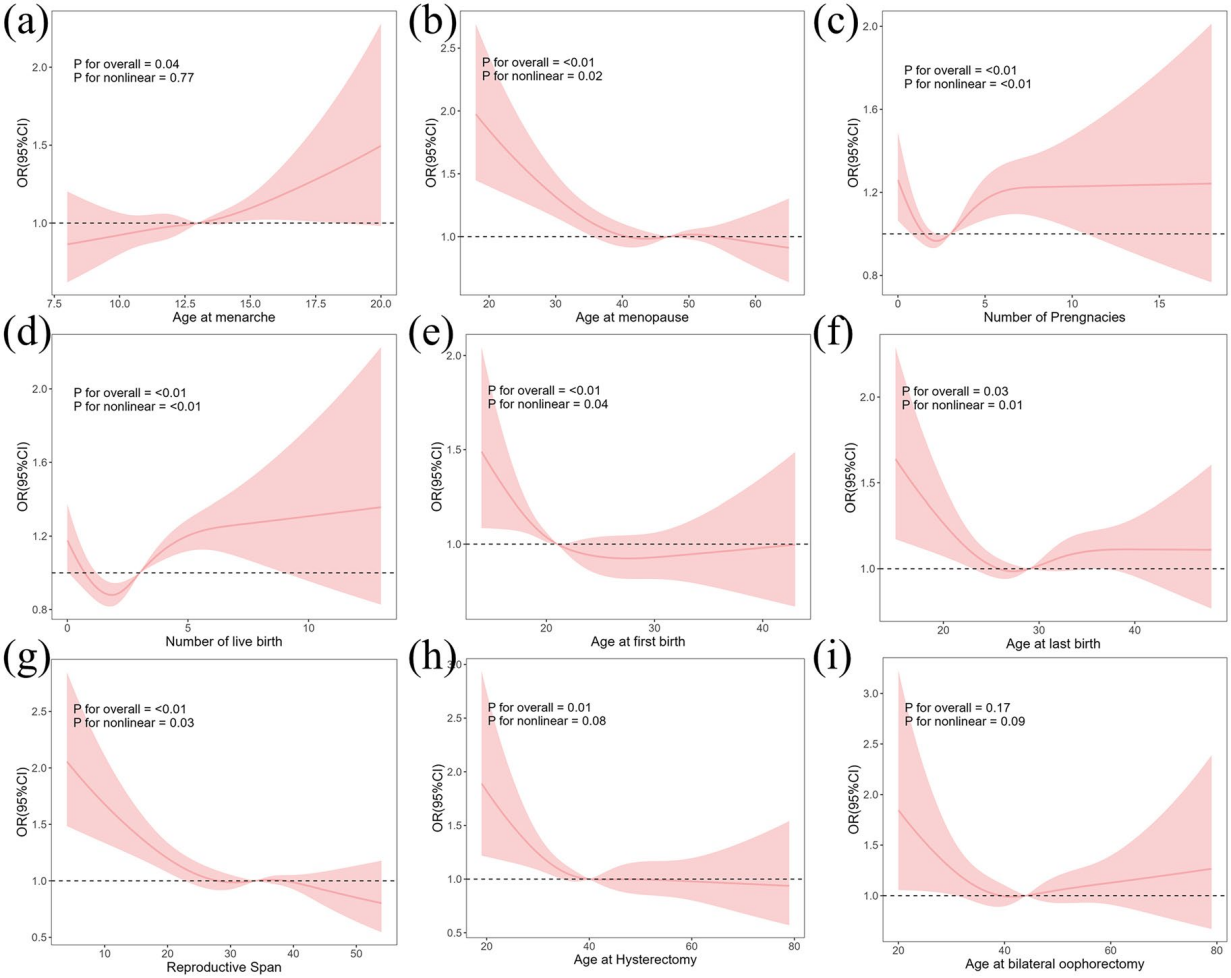
Adjusted associations between continuous reproductive exposures and the prevalence of chronic kidney disease using restricted cubic spline regression models. (a) age at menarche; (b) age at menopause; (c) number of pregnancies; (d) number of live births; (e) age at first birth; (f) age at last birth; (g) reproductive lifespan (h) age at hysterectomy; (i) age at oophorectomy.

All continuous reproductive exposures showed significant overall associations with CKD prevalence in the adjusted RCS regression models (Figure 6). Non-linear associations were observed for most exposures, except for age at menarche, which exhibited an approximately linear relationship (P for non-linearity >0.05).

In sensitivity analyses restricted to NHANES cycles with available gestational diabetes mellitus data, the association between higher parity and CKD was weaker after additional adjustment for GDM. However, the estimates were imprecise and no longer statistically significant, likely due to reduced sample size in these restricted analyses (Table 4).

**Table 4.** Crude and multivariable-adjusted associations between parity and chronic kidney disease, with additional adjustment for gestational diabetes mellitus.

		Prevalence of reproductive exposure^⸿^		Crude model		Adjusted model one^&^		Adjusted model two^¶^	
Reproductive exposures	Categories	CKD group	Non-CKD group	OR (95%CI)	*P-Value*	OR (95% CI)	*P-Value*	OR (95% CI)	*P-Value*
Number of live births	0	0.4 (0.1, 0.7)	0.3 (0.1, 0.5)	1.56 (0.50, 4.88)	0.437	1.56 (0.48, 5.04)	0.454	1.57 (0.48, 5.13)	0.449
	1−2	43.1 (40.0, 46.3)	57.0 (55.1, 58.8)	Ref.		Ref.		Ref.	
	3−4	40.5 (37.9, 43.2)	34.8 (33.3, 36.4)	1.54 (1.32, 1.79)	**<0.001**	1.15 (0.97, 1.37)	0.103	1.15 (0.97, 1.37)	0.107
	≥5	16.0 (14.0, 18.0)	7.9 (6.9, 8.9)	2.67 (2.17, 3.29)	**<0.001**	1.26 (0.99, 1.60)	0.065	1.25 (0.98, 1.60)	0.070

^⸿^ Weighted percentage using NHANES sampling design.

^&^ Adjusted model one included age, body mass index, race/ethnicity, educational attainment, family income-to-poverty ratio, marital status, smoking status, hypertension, and diabetes.

^¶^ Adjusted model two additionally included history of gestational diabetes mellitus to evaluate the robustness of the associations between reproductive factors and CKD after accounting for pregnancy-related metabolic conditions.

CKD: chronic kidney disease; OC: Oral contraceptives; CI: confidence intervals; HRT: Hormone replacement therapy; OR: odds ratio.

## Discussion

In this large, population-based analysis of female participants from NHANES, we found that several reproductive factors were significantly associated with the prevalence of CKD. A younger age at first birth and higher parity were associated with an increased risk of CKD, whereas HRT and OC use were associated with a lower risk. Restricted cubic spline models further demonstrated significant non-linear associations between most continuous reproductive factors, except age at menarche, and CKD prevalence.

Building on prior NHANES-based research, such as the study by Qian et al. (2022), which examined 4,945 postmenopausal women and focused primarily on early natural and surgical menopause, our study extends these findings by including a substantially larger sample of 12,912 women and evaluating a broader spectrum of reproductive factors, including parity, age at first and last live birth, reproductive lifespan, and histories of HRT and OC use. Furthermore, by incorporating restricted cubic spline models, we were able to explore potential non-linear relationships between continuous reproductive exposures and CKD prevalence. These methodological advancements enabled a more comprehensive assessment of how reproductive history influences CKD risk in postmenopausal women, providing novel insights that complement and expand upon previous NHANES-based analyses [20].

Large population-based cohort studies have consistently demonstrated that adverse pregnancy outcomes, including GDM and hypertensive disorders of pregnancy, are associated with a substantially increased long-term risk of CKD in women [13,27,28]. Although pregnancy-related complications have been implicated in long-term pathways linking reproductive history to later CKD risk in longitudinal cohort studies, we did not observe a statistically significant association between self-reported GDM and prevalent CKD in the NHANES sample. Moreover, additional adjustment for GDM did not materially alter the association between parity and CKD prevalence. Several factors may account for these findings. First, NHANES is a cross-sectional survey, and CKD was assessed at a single time point, which may fail to capture the long latency between pregnancy-related metabolic injury and clinically overt CKD. Second, the limited sample size in survey cycles with available GDM data substantially reduced statistical power, particularly for detecting modest effect sizes. Finally, many women with prior GDM may subsequently develop intermediate cardiometabolic conditions, such as hypertension or overt diabetes, which lie on the causal pathway to CKD; adjustment for these downstream factors may have further attenuated direct associations. Importantly, the absence of a statistically significant association in NHANES should not be interpreted as evidence against the biological relevance of pregnancy complications, but rather reflects the inherent limitations of cross-sectional designs and self-reported exposure ascertainment. Beyond these methodological considerations, a conceptual causal framework may further clarify interpretation. Pregnancy-related complications such as GDM may occupy different positions within the pathway linking reproductive history to CKD. Shared pre-pregnancy risk factors (e.g. obesity, insulin resistance, or socioeconomic disadvantage) may confound associations between reproductive exposures and later CKD, whereas GDM may also function as an intermediate variable on the pathway from reproductive exposures to long-term cardiometabolic injury and subsequent CKD. In this setting, statistical adjustment for GDM could attenuate associations if it operates as a mediator rather than a pure confounder. Given the cross-sectional design of NHANES and limited temporal information, we cannot formally distinguish mediation from confounding; therefore, adjusted models should be interpreted as associations under different conditioning strategies rather than definitive causal effects.

In our study, CKD prevalence was higher among women with younger age at first birth and lower among those with more advanced maternal age. These findings are consistent with previous research suggesting that, although pregnancy involves substantial hormonal changes, the observed associations are at least partly attributable to sociodemographic factors [29]. Women who gave birth at a younger age tended to have lower socioeconomic status, poorer overall health, and a higher burden of comorbidities, all of which may contribute to increased CKD risk [30,31]. In contrast, the protective associations of HRT and OC use are more plausibly explained by biological mechanisms related to estrogen exposure. Several molecular mechanisms may underlie the protective effects of estrogen on kidney function. Estrogen can improve renal hemodynamics by modulating the renin–angiotensin–aldosterone system and endothelin-1 signaling, downregulating angiotensin II type 1 receptor, upregulating angiotensin-converting enzyme 2/Ang-(1–7) signaling, and suppressing endothelin-1–mediated vasoconstriction and inflammation. Estrogen also attenuates renal fibrosis and oxidative stress *via* activation of the G protein–coupled estrogen receptor/Sirtuin 1–high mobility group box 1 axis, enhancing antioxidant enzyme expression, and maintaining extracellular matrix homeostasis through regulation of matrix metalloproteinases/tissue inhibitors of metalloproteinases and transforming growth factor-β1 signaling. Furthermore, estrogen influences sex hormone balance through aromatization and feedback on the hypothalamic-pituitary-gonadal axis, which may contribute to sex-specific differences in CKD susceptibility [32,33]. In line with these mechanisms, recent clinical studies have demonstrated that contemporary hormone therapy regimens, including transdermal estrogen and tailored HRT, are associated with improved cardiovascular and metabolic outcomes, which may indirectly support renal protection [34,35]. These findings highlight the translational relevance of mechanistic insights into estrogen-mediated renal protection in postmenopausal women. However, the impact of exogenous estrogen on kidney function has shown conflicting results. We found that both HRT and OC use were associated with a lower risk of CKD, consistent with findings from a 5-year follow-up cohort study of 491 postmenopausal women, which reported that HRT users had a reduced risk of developing albuminuria [11]. In contrast, a longitudinal study of 5,845 postmenopausal women and a UK Biobank analysis both reported an increased risk of CKD associated with ever-use of HRT^[8,12]^. The heterogeneity in previous findings remains difficult to interpret, possibly due to variations in estrogen compound types, delivery methods, co-administration with different progestins, and the initiation window relative to menopause [36].

Our findings indicate that the observed increased prevalence of CKD among women with certain reproductive histories is not only statistically significant but also clinically meaningful, as it may help refine CKD risk stratification in postmenopausal women. Women with higher parity or younger age at first birth may be at increased risk and could benefit from earlier or more frequent kidney function monitoring. Conversely, histories of HRT or OC use, which appear protective, may inform individualized counseling and preventive strategies. Integrating reproductive factors into CKD risk assessment could enhance early detection and guide lifestyle or medical interventions to preserve kidney health. Supporting this, previous studies have reported that reproductive factors, including reproductive period, age at menarche, and age at menopause, were significantly associated with diabetes, metabolic syndrome, and cardiovascular disease, all established risk factors for CKD, highlighting their potential utility in guiding early screening and preventive interventions [9,37]. Collectively, these findings emphasize the translational relevance of reproductive history in identifying women at higher CKD risk and tailoring preventive strategies accordingly.

First, this study evaluated a broad range of reproductive milestones and interventions, and conducted additional sensitivity analyses accounting for pregnancy-related metabolic complications where data were available, allowing a more nuanced assessment of their associations with CKD. Second, the use of NHANES data, collected through a nationally representative survey with rigorous quality control, enhances both the generalizability and reliability of our findings. Third, by incorporating RCS models, we were able to capture potential non-linear associations between reproductive factors and CKD risk, offering insights beyond those obtainable from conventional linear analyses.

Several potential limitations of our study should be acknowledged. First, information on reproductive history and exogenous hormone use was self-reported, which may be subject to recall bias and exposure misclassification, including potential misclassification of menopausal status, potentially attenuating the observed associations. Second, the cross-sectional design of NHANES limits causal inference. Although most reproductive events, such as menopause and childbirth, typically occur before the age when CKD is commonly diagnosed, we cannot entirely rule out reverse causation. We also could not reliably exclude participants with preexisting CKD, assess incident CKD, or evaluate CKD progression over time. In addition, the specific etiology of CKD could not be determined, as detailed information on underlying causes such as diabetic nephropathy, hypertensive nephrosclerosis, or glomerulonephritis was unavailable; therefore, our findings should be interpreted as associations with overall CKD prevalence rather than disease-specific risk. Third, although we adjusted for a wide range of confounders, residual confounding or unmeasured factors cannot be completely excluded. These may include lifestyle factors (e.g. physical activity, dietary intake), use of nephrotoxic medications, cardiovascular disease history, genetic predisposition, or hormone levels. Fourth, we were unable to assess whether the association between HRT use and CKD risk varied by hormone formulation (e.g. estrogen alone vs. estrogen plus progestin) or by timing of initiation relative to menopause. Fifth, information on pregnancy-related complications in NHANES was incomplete. Although GDM was available in selected survey cycles and was examined both as an independent exposure and in sensitivity analyses, data on other important pregnancy complications (preeclampsia and gestational hypertension) were not consistently collected across cycles. Moreover, analyses involving GDM were restricted to a subset of participants, resulting in reduced sample size and statistical power. As a result, the potential contribution of pregnancy complications to the observed associations between reproductive history and CKD may not have been fully captured.

## Conclusions

The prevalence of CKD was significantly associated with several reproductive factors. Younger age at first birth and higher parity were linked to an increased likelihood of CKD, whereas hormone replacement therapy and oral contraceptive use were associated with a lower risk. These findings suggest that reproductive history may contribute to the long-term renal health of women and could be incorporated into CKD risk stratification among postmenopausal populations. Further longitudinal and mechanistic studies are warranted to clarify the causal pathways underlying these associations and to determine whether reproductive history can inform early identification and prevention strategies for CKD in women.

## Data Availability

The datasets analyzed during the current study are publicly available from the NHANES website (https://www.cdc.gov/nchs/nhanes/).
